# Treatment of Primary Axillary Hyperhidrosis with Botulinum Toxin Type A: Our Experience in 50 Patients from 2007 to 2010

**DOI:** 10.5402/2012/702714

**Published:** 2012-10-17

**Authors:** Stefano Scamoni, Luigi Valdatta, Claudia Frigo, Francesca Maggiulli, Mario Cherubino

**Affiliations:** Plastic Surgery Unit, Circolo and Fondazione Macchi Hospital, University of Insubria, Viale Borri 57, 21100 Varese, Italy

## Abstract

*Background*. Local injections of Botulinum toxin type A (BTX-A) are an effective and safe solution for primary bilateral axillary hyperhidrosis. Traditional treatments are often ineffective and difficult to tolerate. This study was performed to assess the efficacy and safety of Botulinum toxin type A in the treatment of these diseases and to evaluate the reliability of patient's subjective rating in the timing of repeat injections. *Methods*. From 2007 to 2008, we included in the study and treated a total of 50 patients, and we used the Minor's iodine test and the hyperhidrosis diseases severity scale as initial inclusion criteria and also for evaluating the followup, comparing to patient's subjective rating. We used also a specific questionnaire to evaluate the level of pain, the onset of the effect, any eventual adverse effect of the treatment, the onset of compensatory hyperhidrosis, and the global grade of satisfaction. The data were analyzed using standard statistical methods. *Results*. 88% of patients were totally satisfied and all patients repeated the treatment during all the study. The symptom-free interval was in median 6 months with an average improving of HDSS of 1.5 points. In 86%, there was a complete accordance between the subjective patient's demand of the repetition of the treatment and the positivity to Minor test and HDSS. No major side effects happened. *Conclusion*. Local injections of Botulinum toxin type A (BTX-A) result in an effective and safe solution for bilateral axillary primary hyperhidrosis for the absence of significant morbidity, side effects, and lack of efficacy or duration. The only defects are the need of repetition of the treatment and relative costs.

## 1. Introduction

Primary hyperhidrosis is an idiopathic and chronic disorder of incontrollable excessive sweating without a recognizable cause [1–4]. It can affect any part of the body, be focal or generalized and commonly it affects the axillae (axillary hyperhidrosis), palms of the hands (palmar hyperhidrosis), the soles of the feet (plantar hyperhidrosis), and the face (facial hyperhidrosis) [5].

This condition causes significant problems both in social and private daily life and affects about 2.5% of the population [6, 7].

Traditional treatments include topical aluminium salts, ionthoforesis, and systematic anticholinergic drugs that are often ineffective, short acting, and difficult to tolerate [1, 8–10]. Surgical procedures such as liposuction, direct excision of the glands, or sympathectomy can have serious risks [11].

Local injections of Botulinum toxin type A (BTX-A) result in an effective and safe solution for primary hyperhidrosis [12–26]. The principal target of BTX-A is the cholinergic nerve endings, and the action is to block the release of acetylcholine from the presynaptic nerve terminal, obtaining a temporary and reversible local chemodenervation [13, 27–30].

The aim of the study was to evaluate the efficacy and the safety of Botulinum toxin A intradermal injection in the treatment of primary axillary hyperhidrosis, investigating the optimal timing of repeat injections, symptoms-free period, and the subjective improving of quality life.

## 2. Patients and Methods

This was a single-center, open-label nonrandomized study with no controls.

The inclusion criteria were adult patients with primary axillary hyperhidrosis that resulted positive to the colorimetric technique of Minor's iodine test and with a 3 or 4 score of hyperhidrosis disease severity scale, without medical conditions such as myasthenia gravis or drugs as aminoglycoside antibiotics that interfere with neuroglandular transmission, infection or skin diseases, and any other concurrent treatments for hyperhidrosis. All subjects were enrolled voluntarily and informed on other alternative methods for treatment.

At the enrolment visit of the study in addition to personal and clinic patient's data recording, was performed the Minor's iodine test and the hyperhidrosis diseases severity scale as initial inclusion criteria and to all patient, after the procedure was given a specific questionnaire, (see attachment 1, in Supplementary Material available online at doi:10.5402/2012/702714), to evaluate the level of pain, the onset of the effect, any eventual adverse effect of the treatment, the onset of compensatory hyperhidrosis, and the global grade of satisfaction.

We choose the Minor's iodine test and hyperhidrosis, diseases severity scale as inclusion criteria and followup tolls for their simplicity and reproducibility. 

The Minor iodine test is a useful technique for clinical evaluation of the hyperhidrosis that shows the effective location of the hyperactive sweat glands to be threaded at the first time or after, during the followup, the eventual residual areas of hyperhidrosis [31, 32].

**Figure 1:**
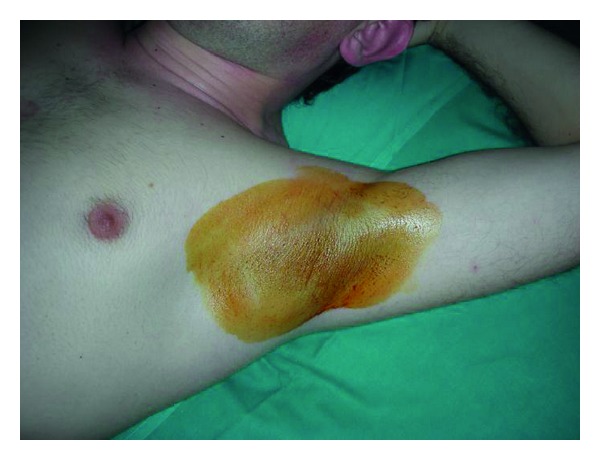
Minor's iodine test, the axilla is painted with a 2% of iodine solution.

**Figure 2:**
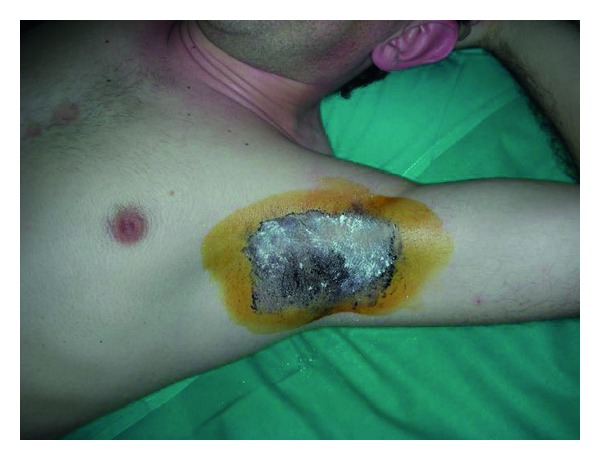
Minor's iodine test, the potato starch powder is applied.

**Figure 3:**
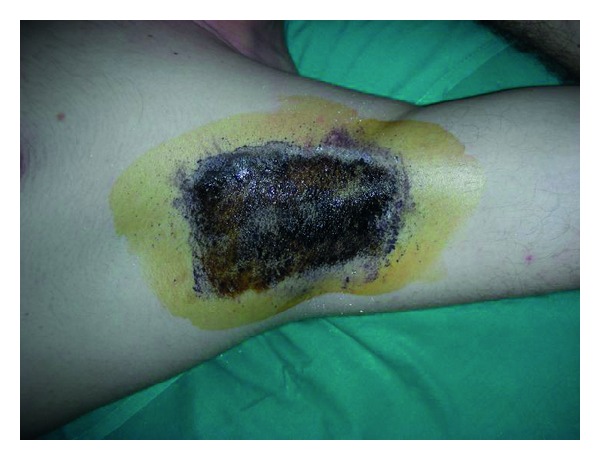
Minor's iodine test, after 15 minutes the presence of sweating is indicated by the onset of a dark-blue color.

It's performed painting with a 2% of iodine solution the axilla and after the solution is dried applying a potato starch powder. Subjects are exposed to a 30°C room temperature and are observed after 10–15 minutes and the presence of sweating is indicated by the onset of a dark-blue color. The borders of the area are marked and the photographs are taken with a 12 cm ruler next to the axilla [32, 33] (Figures 1, 2, and 3).

We did not use the gravimetry and the evaporimetry for their clinical use limitations as complexity of realization and above all intra- and interpatients variability [34].

The HDSS is a disease-specific scale for hyperhidrosis that provides a qualitative measure of the severity of the disease based on how and as the hyperhidrosis affects patient's daily activities [35].

The patient has only to select the statement that best reflects his condition from 4 possible choices.

A score of 1 or 2 indicates a mild or moderate hyperhidrosis and a score of 3 or 4 indicates severe hyperhidrosis [36].

This is a quick diagnostic tool that can be rapidly administered in written format, is easily understood, and requires no aid for completion (Table 1).

The validity and reliability of the HDSS was analyzed and confirmed in other studies that found correlations with the more complex HHIQ, Dermatology Life Quality Index, and mostly with the gravimetric sweat production for the followup, highlighting that a 1-point improvement of the HDSS score corresponds to a 50% of reduction in sweat production and a 2-point improvement corresponds to a 80% of reduction in sweat production [7, 36–38].

From 2007 to 2008, we included in the study and treated a total of 50 patients: 28 females (56%) and 22 males (44%) with a followup, respectively, of 2 years. 

**Figure 4:**
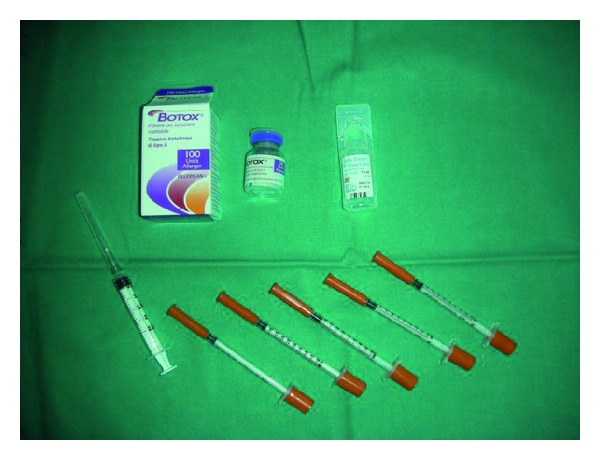
100 U of Botox (Allergan, Inc., Irvine, CA, USA).

**Figure 5:**
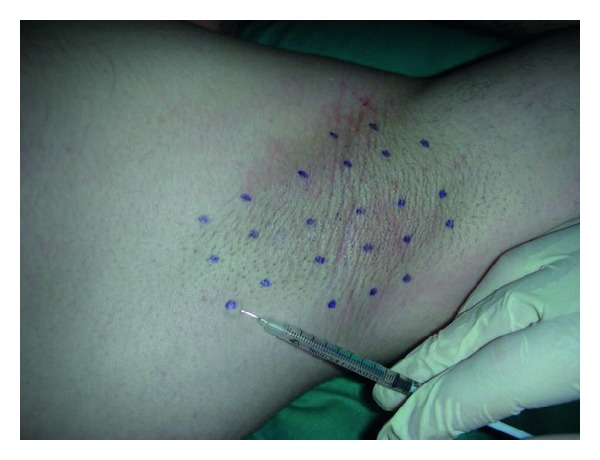
2 cm separated injection sites.

Each patient was treated with 100 U of Botox (Allergan, Inc., Irvine, CA, USA) reconstituted with 2.5 mL of 0.9% sterile saline subdivided into 25 subdermal injections of 2 U 2 cm separated from each other for axilla in the marked area at the minor test (Figures 4–5) and after the procedure the questionnaire was given (Attachment 1). No local anaesthesia was needed.

The first follow-up visit was one month later with the receiving of the questionnaire and the repetition of the HDSS and the minor test for evaluating the effect and treating eventual residual areas of hyperhidrosis.

The other follow-up visits were fixed at 6, 8, 10, and 12 months after the first treatment with the repetition of the HDSS and the minor test to individuate the return of the hyperhidrosis and the repetition of the treatment was performed when first inclusion criteria (minor test positivity and 3 or 4 score of HDSS) were met.

After the second treatment, the followup restarted from zero as for the first treatment and so on for next treatments.

## 3. Results

From 2007 to 2008, we included in the study and treated a total of 50 patients affected by bilateral primary axillary hyperhidrosis: 28 females (56%) and 22 males (44%) with a followup of 2 years. All patients were below the age of 52 years with a median of 28 years (Table 2).

All of them repeated the treatment till 2010 with a duration of symptom relief ranging from 4 to 12 months, with a median of 6 months and a number of repeated treatments from 2007 to 2010 ranging from 3 to 5, with a median of 4 times.

A total of 44 patients (88%) gave a satisfaction grade from 80 to 100% indicating a complete satisfaction, five patients (10%) gave a grade from 70 to 80% indicating a more than sufficient satisfaction, and 1 patient (2%) had minor complication with pain in the first days and this gave a moderate satisfaction grade.

Side effects were negligible including bruising and nuisance for maximum two days after injection in 9% of patients and transient and slight compensatory hyperhidrosis of the trunk in 6% of patients. We did not have any major side effect (Table 3).

We reported a reduction of the HDSS at 4 weeks after the injections of tree points in one patient (2%), of two points in 34 patients (68%), and of one point in 15 patients (30%), and we needed to treat focal residual of sweating areas only in four patients.

In the 86%, there was a complete accordance between subjective patient's demand of repetition of the treatment (with HDSS score of 3 or 4) and the achievement of the other inclusion criteria (positivity to the Minor test); in the remaining 14%, the personal demand of patient anticipated the positivity of Minor test from 1 to 3 months with a median approximately of 2 months. 

In all repeated treatments, we did not observe any lack of efficacy or reduction of the duration of symptom relief.

## 4. Discussion

Axillary hyperhidrosis causes considerable emotional and social problems affecting the daily life.

Those patients have evident impediments both in social and private life, they wet their clothes in few minutes also in the winter time, and they have to change their clothes several time in a day, above the discomfort they also have to support costs for cleaning and changing their clothes [39].

Previous therapies are either ineffective or associated to unacceptable morbidity or side effects.

The use of toxin Botulinum toxin type A seems to overcome the morbidly problem, given a safe, fast, and easy to do treatment.

We found a complete responder rate, with a good-optimal grade of global satisfaction and all the patients treated returned for the repetition of the treatment.

We also found a significant correspondence (86%) between HDSS and Minor test and the subjective needs for the first treatment or the repetition, confirming the optimal utility in clinical practice of these methods and attributing a large importance to subjective patient's opinion.

## 5. Conclusion

We believe that, Botulinum toxin type A is a safe, easy, and fast procedure for the treatment of primary bilateral axillary hyperhidrosis also in secondary cases, not only in the series that we present [13, 33]. The main problem is still the relative cost but considering the absence of significant morbidity, side effects, and lack of efficacy or duration, we believe that local injections of Botulinum toxin type A (BTX-A) result in an effective and safe solution.

## Supplementary Material

Attachment 1: Specific questionnaire to evaluate the level of pain, the onset of the effect, its duration and the global grade of satisfaction.

## Figures and Tables

**Table 1:** Hyperhidrosis disease severity scale.

Hyperhidrosis disease severity scale	
My axillary sweating is never noticeable and never interferes with my daily activities	Score 1
My axillary sweating is tolerable but sometimes interferes with my daily activities	Score 2
My axillary sweating is barely tolerable and frequently interferes with my daily activities	Score 3
My axillary sweating is intolerable and always interferes with my daily activities	Score 4

**Table 2:** Patient demographics.

Patient demographics	Number (%)
Mean age	28
Range	18–52
Sex	
Male	22 (44%)
Female	28 (56%)

**Table 3:** Results.

Satisfaction grade at one month	Number (%)
91–100%	10 (20%)
81–90%	34 (68%)
71–80%	5 (10%)
61–70%	1 (2%)

Reduction of HDSS at four weeks	
1 point	1 (2%)
2 points	34 (68%)
3 points	15 (30%)

Duration of symptom relief	
4–6 months	40 (80%)
6–8 months	6 (12%)
8–10 months	3 (6%)
10–12 months	1 (2%)

Accordance between the subjective patient's demand and HDSS-Minor test	
Complete	43 (86%)
1 month	2 (4%)
2 months	4 (8%)
3 months	1 (2%)

Side effects	
No side effects	38 (76%)
Bruising and nuisance	9 (8%)
Compensatory trunk hyperhidrosis	3 (6%)
