# Supplementary material for: Treatment of Primary Axillary Hyperhidrosis with Botulinum Toxin Type A: Our Experience in 50 Patients from 2007 to 2010
Source: ISRN Dermatol. 2012 Oct 17;2012:702714. doi: 10.5402/2012/702714 (PMC3483720; doi:10.5402/2012/702714)
Supplement: Supplementary file 1 — Attachment 1: Specific questionnaire to evaluate the level of pain, the onset of the effect, its duration and the global grade of satisfaction. [file 702714.f1.docx]

How was the pain during the injections?

| 0 | 1 | 2 | 3 | 4 | 5 | 6 | 7 | 8 | 9 | 10 |
| --- | --- | --- | --- | --- | --- | --- | --- | --- | --- | --- |

How was the pain in the first days after the injections?

| 0 | 1 | 2 | 3 | 4 | 5 | 6 | 7 | 8 | 9 | 10 |
| --- | --- | --- | --- | --- | --- | --- | --- | --- | --- | --- |

In which day after the injections compared the effect?

| 0 | 1 | 2 | 3 | 4 | 5 | 6 | 7 | 8 | 9 | 10 |
| --- | --- | --- | --- | --- | --- | --- | --- | --- | --- | --- |
| 11 | 12 | 13 | 14 | 15 | 16 | 17 | 18 | 19 | No | effect |

Have you had any side effect in the days after the injections? If yes which?

| No | Yes, which………...………………………………………………………………………………… |
| --- | --- |

At four weeks after the injections which was for you the degree of sweating in percentage?

| 0% | 10% | 20% | 30% | 40% | 50% | 60% | 70% | 80% | 90% | 100% |
| --- | --- | --- | --- | --- | --- | --- | --- | --- | --- | --- |

At four weeks after the injections did compared any compensatory sweating? If yes where?

| No | Yes, where………...………………………………………………………………………………… |
| --- | --- |

Which is your global grade of satisfaction?

| 0% | 10% | 20% | 30% | 40% | 50% | 60% | 70% | 80% | 90% | 100% |
| --- | --- | --- | --- | --- | --- | --- | --- | --- | --- | --- |

Would you recommend this treatment to other people? If no why?

| Yes | No, why….………………………………………………………………………………………… |
| --- | --- |

Have you got any comment or suggestion? If yes which?

| No | Yes, which………...………………………………………………………………………………… |
| --- | --- |

Attachment 1: specific questionnaire
